# Optimal dose of tirzepatide for type 2 diabetes mellitus: A meta-analysis and trial sequential analysis

**DOI:** 10.3389/fcvm.2022.990182

**Published:** 2022-08-31

**Authors:** Yunfeng Yu, Gang Hu, Shuang Yin, Xinyu Yang, Manli Zhou, Weixiong Jian

**Affiliations:** ^1^College of Chinese Medicine, Hunan University of Chinese Medicine, Changsha, China; ^2^The First Affiliated Hospital of Hunan University of Chinese Medicine, Changsha, China

**Keywords:** tirzepatide, dose, type 2 diabetes mellitus, meta-analysis, trial sequential analysis

## Abstract

**Objective:**

The purpose of this study is to evaluate the optimal dose of tirzepatide (TZP) for the treatment of type 2 diabetes mellitus (T2DM) by meta-analysis and trial sequential analysis (TSA).

**Methods:**

Clinical trials of TZP for T2DM were obtained by searching 8 databases with a time limit from database creation to May 2022. Mean differences (MD) and 95% confidence intervals (95%CI) were used for continuous variables, and relative risk (RR) and 95%CI were used for dichotomous variables.

**Results:**

Compared with TZP 5 mg, meta-analysis showed that TZP 10 mg significantly reduced glycosylated hemoglobin type A1c (HbA1c) (MD −0.24, 95%CI −0.31~-0.17, *P* < 0.00001), fasting serum glucose (FSG) (MD −5.82, 95%CI −8.35~-3.28, *P* < 0.00001) and weight (MD −2.47, 95%CI −2.95~-1.98, *P* < 0.00001), and TZP 15 mg significantly reduced HbA1c (MD −0.37, 95%CI −0.44~-0.29, *P* < 0.00001), FSG (MD −8.52, 95%CI −11.07~-5.98, *P* < 0.00001) and weight (MD −4.63, 95%CI −5.45~-3.81, *P* < 0.00001). Compared with TZP 10 mg, TZP 15 mg dramatically reduced HbA1c (MD −0.12, 95%CI −0.19~-0.05, *P* = 0.001), FSG (MD −2.73, 95%CI −5.29~-0.17, *P* = 0.04) and weight (MD −2.18, 95%CI −2.67~-1.70, *P* < 0.00001). The TSA indicated that the benefits observed in the current information set were conclusive, except for the FSG of “TZP 15 mg vs. TZP 10 mg”. In terms of safety endpoints, meta-analysis revealed that there was no significant difference in the serious adverse events (AEs), major adverse cardiovascular events-4 (MACE-4), cardiovascular death, hypertension, cancer and hypoglycemic of the three dose groups of TZP. Compared with TZP 5 mg, TZP 10 mg increased total adverse events (RR 1.06, 95%CI 1.01~1.11, *P* = 0.03) and gastrointestinal (GI) AEs (RR 1.17, 95%CI 1.03~1.33, *P* = 0.02), and TZP 15 mg increased total AEs (RR 1.10, 95%CI 1.05~1.15, *P* = 0.0001). There were no significant differences in total AEs and GI AEs for TZP 15 mg compared to TZP 10 mg. The TSA demonstrated that the total AEs of “TZP 15 mg vs. TZP 5 mg” were conclusive.

**Conclusions:**

TZP 15 mg >TZP 10 mg > TZP 5 mg in terms of lowering glycemia and reducing weight. TZP 5 mg > TZP 10 mg = TZP 15 mg in terms of safety. On this basis, we recommend TZP 5 mg as the first-choice dose for patients with T2DM to minimize AEs while reducing glycemia and weight. If patients cannot effectively control their glycemia after taking TZP 5 mg, it is recommended to take TZP 15 mg directly to achieve the best effect of glycemic reduction. However, most of the included studies have the background of basic medication, the independent efficacy and safety of different doses of TZP still need to be tested.

**Systematic review registration:**

Unique Identifier: CRD42022341966.

## Introduction

Type 2 diabetes mellitus (T2DM) is a chronic progressive metabolic disease characterized by pancreatic β-cell dysfunction as well as insulin resistance (1). According to statistics, approximately 1 in 11 adults worldwide has diabetes, 90% of whom have T2DM (2). The chronic and persistent hyperglycemic state of T2DM increases the risk of cardiovascular disease, nephropathy and retinopathy (3, 4) which significantly increases global mortality as well as disability (2), and T2DM has become one of the most significant risk factors threatening human health (5). The core aspect of T2DM is insulin resistance formed by a combination of multiple factors, and obesity is one of the important causes of insulin resistance (6). Glycemia and glycosylated hemoglobin type A1c (HbA1c) have been reported to be negatively correlated with the amount of adiposity in patients with T2DM (7). It has been revealed that weight loss is an important predictor of T2DM delay (8). A moderate weight loss can significantly improve glycemic control and reduce the risk factors of cardiometabolism in patients with T2DM (9). Thus, aggressive glycemic control and weight loss are essential in the treatment of T2DM before irreversible damage of islet β cells (10). Although insulin and glucagon like peptide-1 receptor agonist (GLP-1 RA) exert a good role in glycemic control in T2DM, unfortunately insulin has the risk of triggering hypoglycemia (11). Moreover, the efficacy of weight loss of both insulin and GLP-1 RA is not satisfactory (12, 13), and these factors limit the clinical efficacy of insulin and GLP-1 RA. The treatment of T2DM urgently requires a new drug that can achieve effective glycemic reduction and weight loss.

The latest study indicates that tirzepatide (TZP) has a promising application as a novel hypoglycemic drug in the treatment of T2DM (14). TZP is a dual GLP-1 RA and gastric inhibitory polypeptide receptor agonist (GIP RA) (15), which can stimulate insulin secretion by dual activation of glucagon like peptide-1 receptor (GLP-1R) and gastric inhibitory polypeptide receptor (GIPR) in the presence of hyperglycemic state (16) and improve glycemic control in T2DM. In addition, glucagon like peptide-1 (GLP-1) promotes weight loss by delaying gastric emptying, reducing appetite, and inhibiting meals (12, 17). GIPR also stimulates the secretion of glucagon in hypoglycemic states and reduces the occurrence of hypoglycemia (18). TZP has been reported to lower glycemia and reduce weight significantly better than insulin and GLP-1RA (14), with the additional benefit of decreasing metabolites such as triglycerides and lipoproteins (19), and is expected to be a preferred strategy for the treatment of T2DM. The meta-analyses published by Karagiannis et al. (20) and Bhagavathula et al. (21) compared the efficacy and safety of TZP with placebo, insulin and GLP-1 RA in the treatment of T2DM, which confirmed that the hypoglycemic effect of TZP was significantly better than that of placebo, insulin and GLP-1RA. Unfortunately, they did not make an internal comparison of different doses of TZP, and it is unclear the optimal dose and dose adjustment strategy of TZP for the treatment of T2DM. Therefore, in this study, we used three mainstream doses of TZP 5 mg, 10 mg and 15 mg to evaluate the relative efficacy and safety of different doses of TZP for the treatment of T2DM by using meta-analysis and trial sequential analysis (TSA), in order to provide an evidentiary basis for the selection of clinical doses.

## Methods

This study strictly followed the systematic review and meta-analysis methodology of the Preferred Reporting Items for Systematic reviews and Meta-Analyses (PRISMA) (22).

### Literature search

The databases of China National Knowledge Infrastructure (CNKI), China Biology Medicine (CBM), VIP, Wanfang, Embase, PubMed, the Cochrane Library, and Web of Science were searched for clinical studies of TZP for T2DM, all with a time limit from database creation to May 2022. English subject terms included tirzepatide, type 2 diabetes mellitus, and Chinese subject terms included tirzepatide, erxing tangniaobing (the Chinese name of type 2 diabetes mellitus). On the basis of the subject terms, the Chinese free words were expanded with CNKI and CBM databases, and the English free words were expanded with MeSH database. The subject terms and free terms were then combined for the search.

### Inclusion and exclusion criteria

The inclusion criteria are shown below. (1) Study design: Randomized controlled trial. (2) Participants: The basic diagnosis of type 2 diabetes mellitus was met (23). (3) Intervention: Patients in the experimental and control groups were treated with different doses of TZP. (4) Outcomes: HbA1c, fasting serum glucose (FSG) and weight were selected as efficacy endpoints, and total adverse events (AEs), serious AEs, gastrointestinal (GI) AEs, major adverse cardiovascular events-4 (MACE-4), cardiovascular death, hypertension, cancer and hypoglycemic events were selected as safety endpoints. The total AEs refer to a series of TZP-related discomfort symptoms or changes in laboratory indicators that occurred during the study period from the first dose of the drug.

The exclusion criteria were as follows. (1) Studies such as reviews, animal experiments, and case reports. (2) Studies published repeatedly. (3) Studies published in abstract form. (4) Studies with incomplete or unclear data. (Studies that did not provide means and standard deviations for continuous variables or did not provide events and totals for dichotomous variables, and for which these data could not be obtained by conversion).

### Literature screening, statistics and risk of bias

In the initial step, the base literature retrieved from each database was imported into Endnote X9, then duplicates were eliminated in turn, and the literature was eliminated based on the inclusion criteria after reading the title and abstract, and reviewing the full text to finalize the included literature. In the next step, the included literature was categorized and organized to extract basic characteristics such as authors, year, sample size, mean age, sex ratio, intervention, and duration of treatment, which were entered into a statistical table of information. In the final step, the risk of bias was assessed in accordance with the requested entries by using the Cochrane Risk of Bias Assessment Tool. All the above work was carried out independently by two investigators, and any disagreement was decided by a third investigator.

### Statistical analysis

Meta-analysis was conducted using Revman5.3. And relative risk (RR) and 95% confidence intervals (95% CI) were employed as effect sizes for dichotomous variables. Mean differences (MD) and 95% CI were utilized as effect sizes for continuous type variables. Heterogeneity was performed by *I*^2^ test and Q test. If *I*^2^ < 50% and *P* > 0.1, the heterogeneity was small and fixed-effects model analysis was carried out. Otherwise, random effects model analysis was used. Sensitivity analysis was used for indicators with significant heterogeneity, whereby the remaining studies were combined for analysis after excluding one study at a time. If there was no noticeable change in the continuous variables obtained from each combined analysis, the results were suggested to be robust.

TSA0.9.5.10Beta software was applied to perform the TSA. If the cumulative Z value crossed the required information size or TSA threshold, the original result was conclusive. Publication bias was evaluated using Stata15.0 software. If the funnel plot revealed that the scatter on both sides is essentially symmetric and the Harbord regression displayed *P* > 0.1, there was no publication bias. GRADEpro3.6 software was utilized to evaluate the quality of evidence, and the method of evaluation was based on the GRADE evidence evaluation guidelines.

## Results

### Study selection

**Figure 1:**
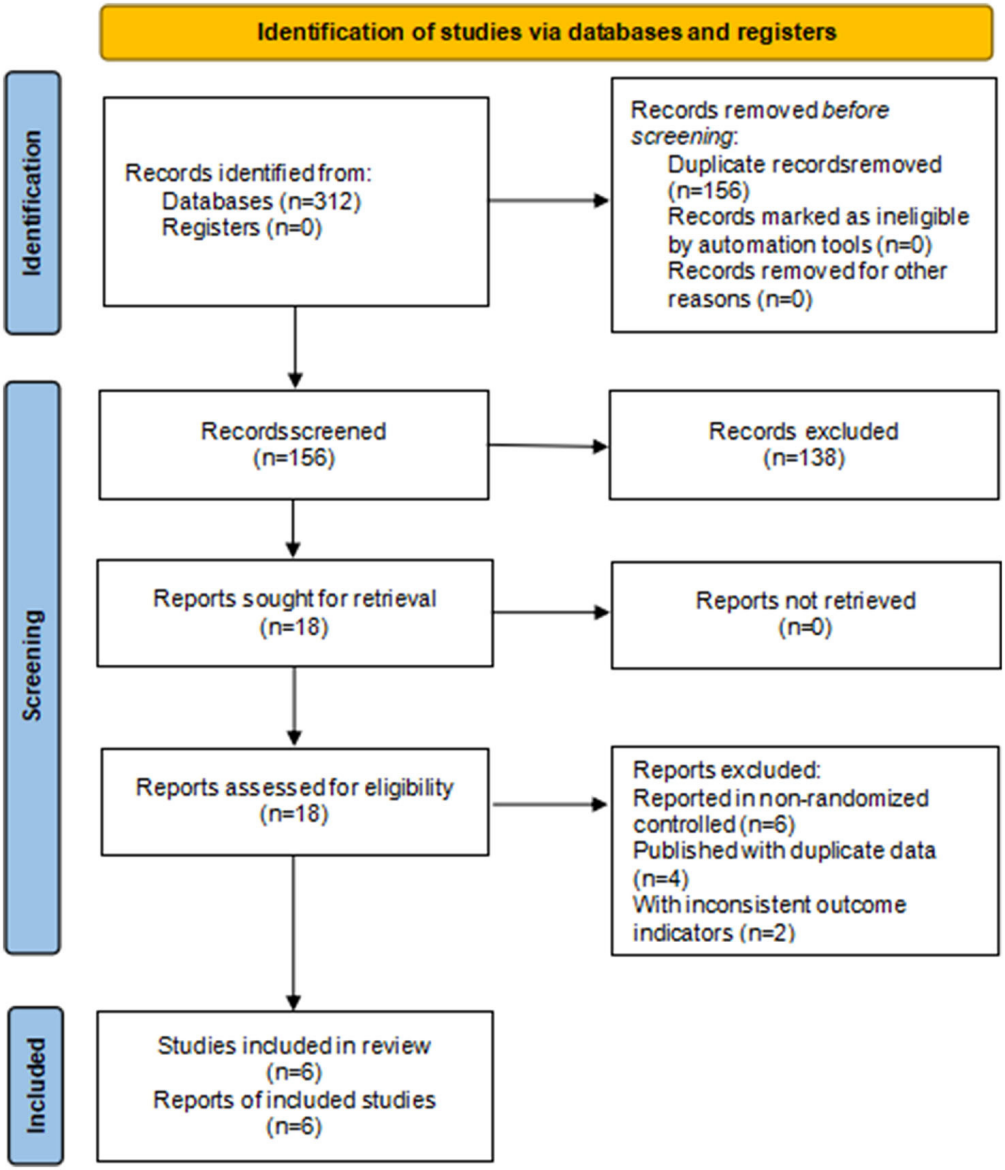
Study flow diagram.

A total of 312 studies were obtained from the search. The 156 duplicates were eliminated. One hundred and twenty eight studies were eliminated after reading the titles and abstracts. Twenty two articles studies were excluded after reading the full text. The reasons for full-text rejection were as follows: those published in abstract form, those published with duplicate data, and those with inconsistent outcome indicators. Six studies were finally included (24–29). As shown in Figure 1.

### Study characteristics

A total of six clinical studies were included (24–29) with a total sample size of 4,358 cases. Except for Rosenstock2021 (24) in which TZP was used alone, the remaining five studies applied the basal medication in combination. Among them, Dahl2022 (25) and Frias2018 (29) used glargine insulin and metformin as their basal medications, respectively, while the remaining three studies (26–28) elaborated on having a background of stable usage of hypoglycemic agents. A total of 1,449 cases of TZP 5 mg, 1,448 cases of TZP 10 mg and 1,461 cases of TZP 15 mg were covered. The research centers were located in five continents, including North America, South America, Europe, Asia, and Oceania. All six studies used the change of HbA1c as the primary efficacy endpoint and the change of FSG and weight as secondary efficacy endpoints. The basic characteristics of the included studies are shown in Table 1.

**Table 1:** Characteristics of included studies.

**Author name**	**Research center**	**Patient number**	**treatment Duration (weeks)**	**Disease duration (years)**	**Intervention**	**Number randomized**	**HbA1c (%)**	**Body weight (kg)**	**Age (Years)**	**Male *N*/%**
Rosenstock (24)	India, Japan, Mexico, USA	363	40	4.6	TZP 5 mg	121	8.0	87.0	54.1	56/46
				4.9	TZP 10 mg	121	7.9	86.2	55.8	72/60
				4.8	TZP 15 mg	121	7.9	85.4	52.9	63/52
Dahl (25)	USA, Japan, Czech Republic, Germany, Poland, Slovakia, Puerto Rico, Spain	355	40	14.1	TZP 5 mg	116	8.3	95.5	62	61/53
				12.6	TZP 10 mg	119	8.3	95.4	60	72/61
				13.7	TZP 15 mg	120	8.2	96.2	61	65/54
Del (26)	Argentina, Australia, Brazil, Canada, Greece, Israel, Mexico, Poland, Romania, Russia, Slovakia, Spain, Taiwan, USA	995	52	9.8	TZP 5 mg	329	8.5	90.3	62.9	198/60
				10.6	TZP 10 mg	328	8.6	90.6	63.7	209/64
				10.4	TZP 15 mg	338	8.5	90.0	63.7	203/60
Ludvik (27)	Argentina, Austria, Greece, Hungary, Italy, Poland, Puerto Rico, Romania, South Korea, Spain, Taiwan, Ukraine, USA	1,077	52	8.5	TZP 5 mg	358	8.2	94.4	57.2	200/56
				8.4	TZP 10 mg	360	8.2	93.8	57.4	195/54
				8.5	TZP 15 mg	359	8.2	94.9	57.5	194/54
Frias (28)	USA, UK, Argentina, Australia, Brazil, Canada, Israel, Mexico,	1,409	40	9.1	TZP 5 mg	470	8.3	92.5	56.3	205/43
				8.4	TZP 10 mg	469	8.3	94.8	57.2	238/50
				8.7	TZP 15 mg	470	8.3	93.8	55.9	214/45
Frias (29)	Poland, Slovakia, Puerto Rico, USA	159	26	8.9	TZP 5 mg	55	8.2	92.8	57.9	34/62
				7.9	TZP 10 mg	51	8.2	92.7	56.5	30/59
				8.5	TZP 15 mg	53	8.1	89.1	56.0	22/42

### Risk of bias assessment

**Figure 2:**
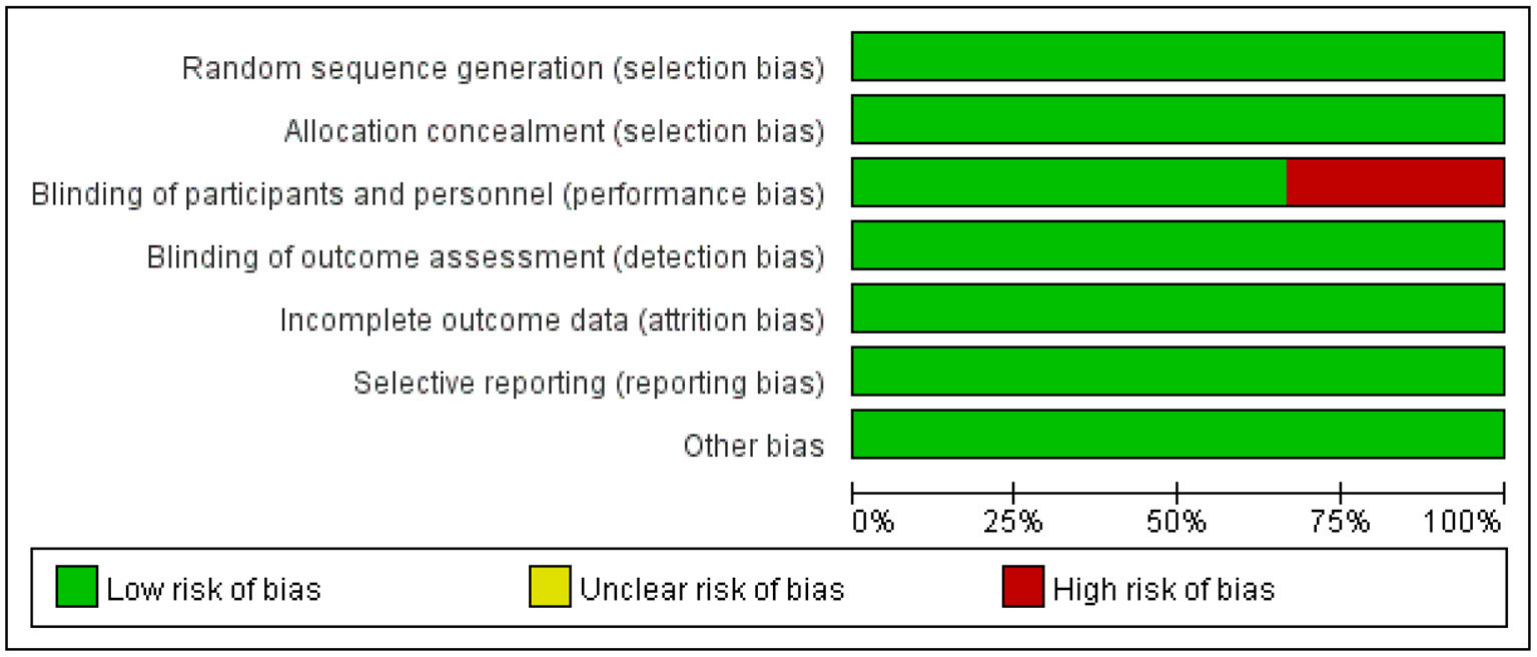
Risk of bias graph.

In the six included studies, Del 2021 (26) and Ludvik 2021 (27) were judged to be at high risk of blinding of participants and personnel because they did not use placebo blinding, with low risk of bias in the remaining areas. The risk of bias of the included studies is shown in Figure 2.

### Efficacy endpoints

#### HbA1c

**Figure 3:**
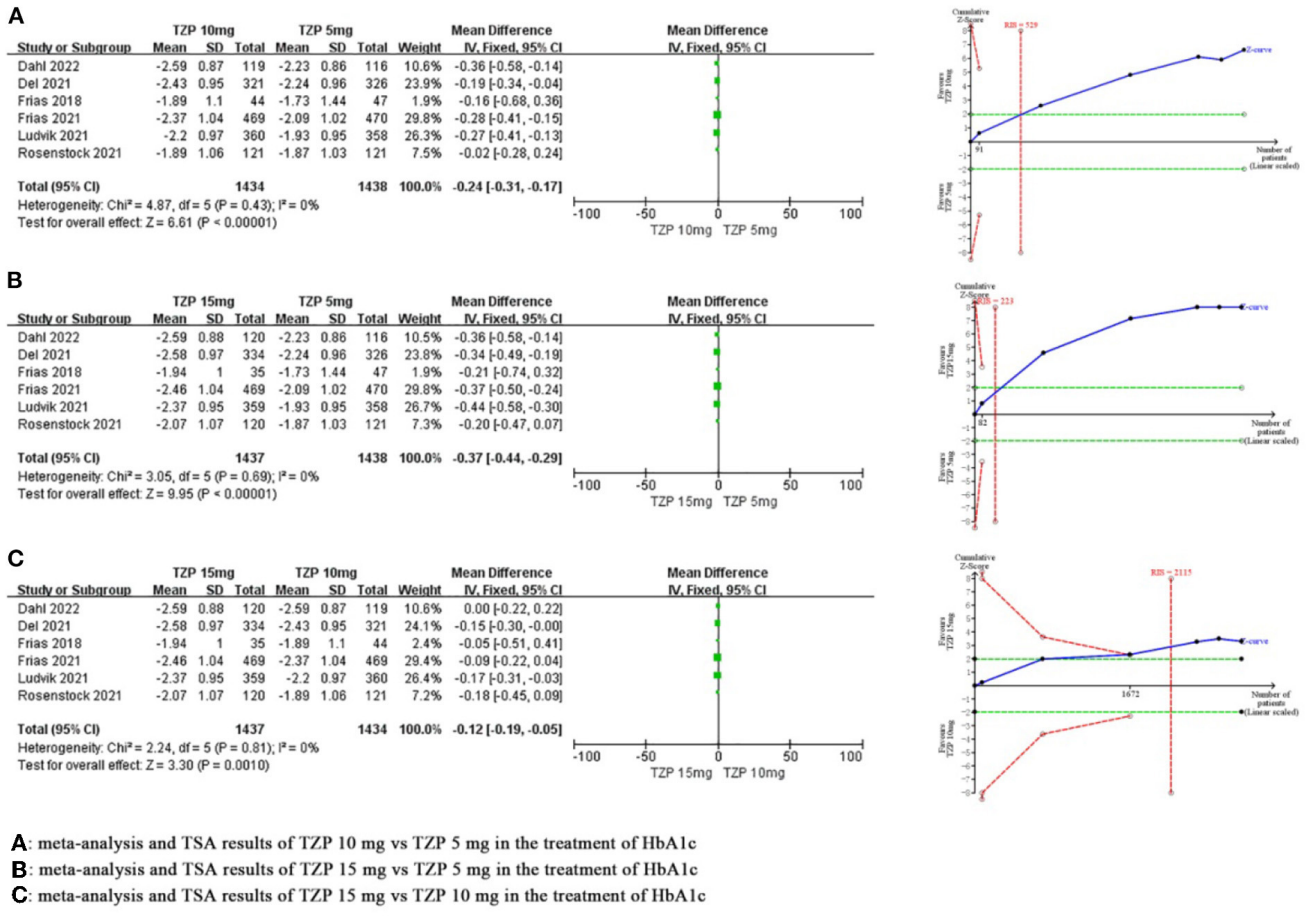
Meta-analysis and TSA results of different doses of TZP for HbA1c.

Compared with TZP 5 mg, meta-analysis demonstrated that the TZP 10 mg could significantly reduce HbA1c by 0.24% (MD −0.24, 95%CI −0.31~-0.17, *P* < 0.00001), and TSA showed that the cumulative Z value crossed the required information size in the second study (RIS = 529), which revealed that this benefit observed for the current information set were conclusive. Compared with TZP 5 mg, meta-analysis demonstrated that the TZP 15 mg could significantly reduce HbA1c by 0.37% (MD −0.37, 95%CI −0.44~-0.29, *P* < 0.00001), and TSA showed that the cumulative Z value crossed the required information size in the second study (RIS = 223), which revealed that this benefit observed for the current information set were conclusive. Compared with the TZP 10 mg, meta-analysis demonstrated that the TZP 15 mg was able to significantly reduce HbA1c by 0.12% (MD −0.12, 95%CI −0.19~-0.05, *P* = 0.001), and TSA showed that the cumulative Z value crossed the required information size in the fourth study (RIS = 2,115), which revealed that this benefit observed for the current information set were conclusive. The GRADE evaluation showed there were no serious risk of bias, inconsistency, indirectness and imprecision, and the quality of evidence for each of these indicators was high. As shown in Figure 3.

#### FSG

**Figure 4:**
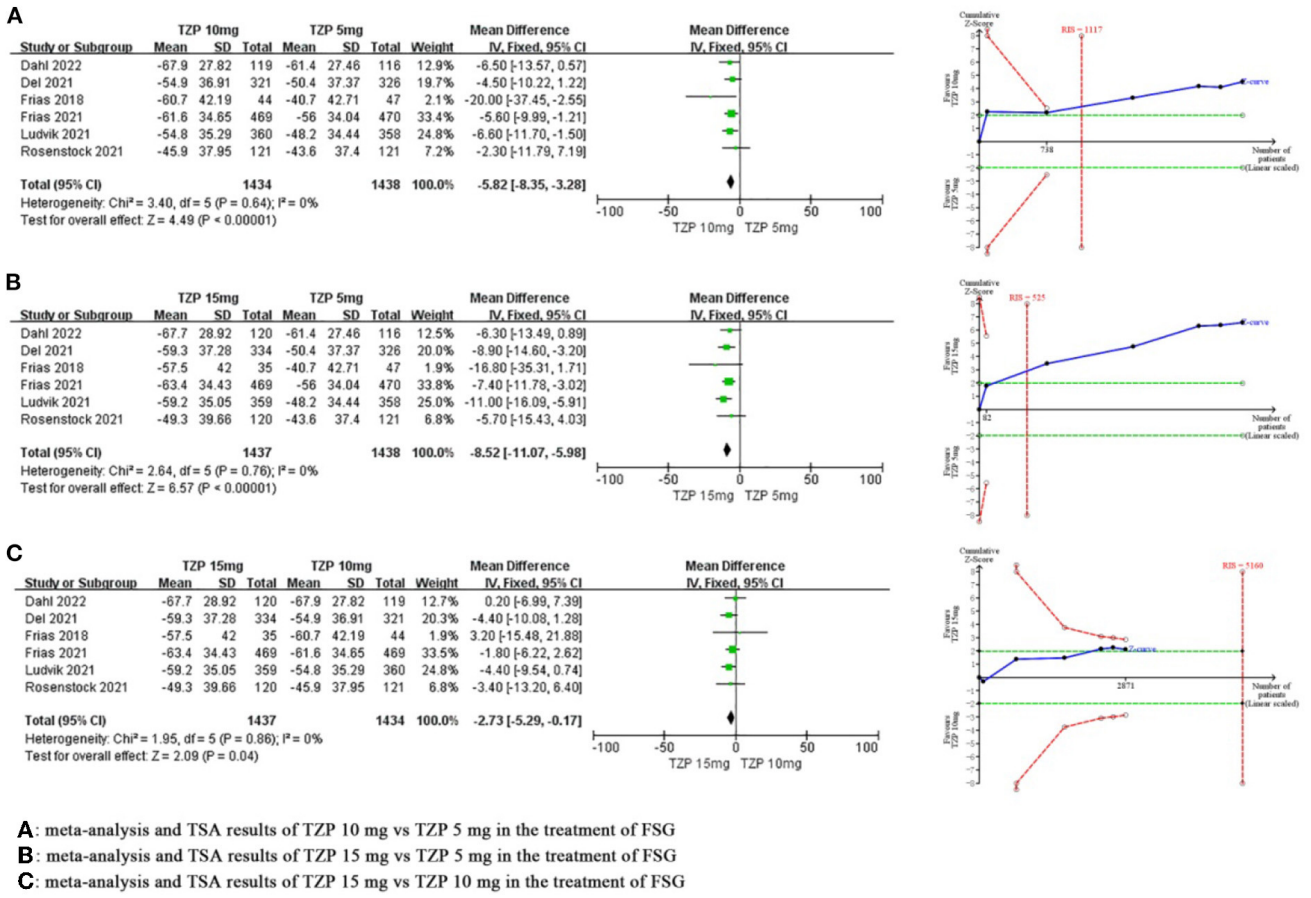
Meta-analysis and TSA results of different doses of TZP for FSG.

Compared with TZP 5 mg, meta-analysis revealed that TZP 10 mg significantly reduced FSG by 5.82 mg/dl (MD −5.82, 95%CI −8.35~-3.28, *P* < 0.00001), and TSA showed that the cumulative Z value crossed the required information size in the third study (RIS = 1,117), which suggested that the benefits observed in the current information set were conclusive. Compared with TZP 5 mg, meta-analysis revealed that TZP 15 mg significantly reduced FSG by 8.52 mg/dl (MD −8.52, 95%CI −11.07~-5.98, *P* < 0.00001), and TSA showed that the cumulative Z value crossed the required information size in the second study (RIS = 525), which suggested that the benefits observed in the current information set were conclusive. Compared with TZP 10 mg, meta-analysis revealed that TZP 15 mg significantly reduced FSG by 2.73 mg/dl (MD −2.73, 95%CI −5.29~-0.17, *P* = 0.04), and TSA showed that the cumulative Z value had not reached the required information size (RIS = 5,160) or TSA boundary value, which suggested the benefits needed to be confirmed by more research. The GRADE evaluation displayed high quality of evidence for all indicators except for the FSG of “TZP 15 mg vs. TZP 10 mg”, which had moderate quality of evidence. As shown in Figure 4.

#### Weight

**Figure 5:**
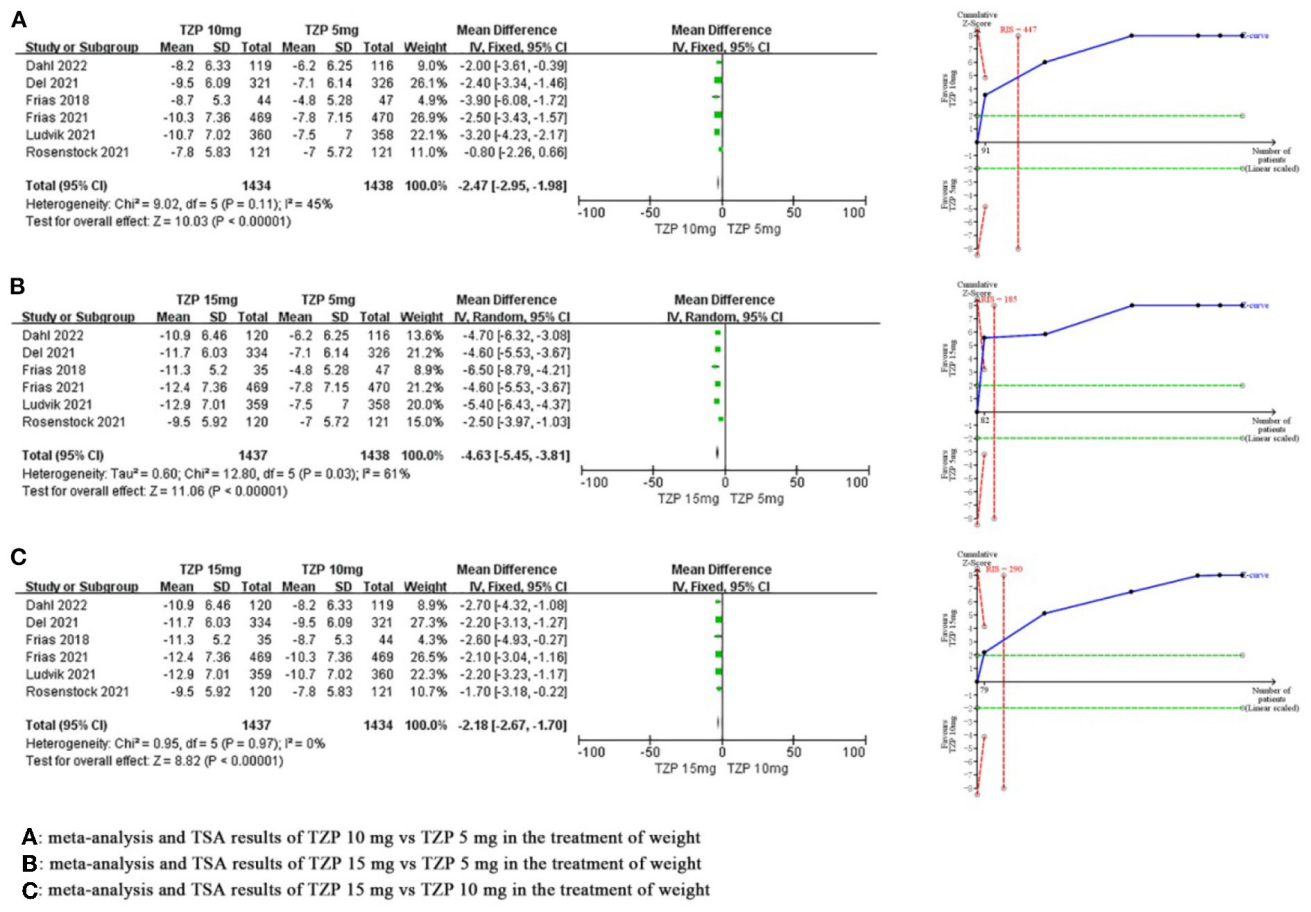
Meta-analysis and TSA results of different doses of TZP for weight.

Compared with TZP 5 mg, meta-analysis showed that TZP 10 mg significantly reduced weight by 2.47 kg (MD −2.47, 95%CI −2.95~-1.98, *P* < 0.00001), and TSA showed that the cumulative Z value crossed the required information size in the second study (RIS = 447), which suggested that the benefits observed in the current information set were conclusive. Compared with TZP 5 mg, meta-analysis showed that TZP 15 mg significantly reduced weight by 4.63 kg (MD −4.63, 95%CI −5.45~-3.81, *P* < 0.00001), and TSA showed that the cumulative Z value crossed the TSA boundary value in the first study (RIS = 185), which suggested that the benefits observed in the current information set were conclusive. Compared with TZP 10 mg, meta-analysis showed that TZP 15 mg significantly reduced weight by 2.18 kg (MD −2.18, 95%CI −2.67~-1.70, *P* < 0.00001), and TSA showed that the cumulative Z value crosses the required information size in the second study (RIS = 290), which suggested that the benefits observed in the current information set were conclusive. Sensitivity analysis showed that the heterogeneity of both “TZP 10 mg vs. TZP 5 mg” and “TZP 15 mg vs. TZP 5 mg” was derived from Rosenstock2021. There was no significant change in the effect size after removing this study, and the differences were still statistically significant, suggesting that the results were robust. The GRADE evaluation revealed that the quality of evidence for all indicators was high, with the exception of weight for “TZP 15 mg vs. TZP 5 mg”, which had a moderate quality of evidence. As shown in Figure 5.

### Safety endpoints

**Figure 6:**
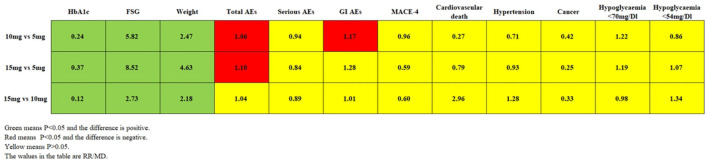
Comparison of each outcome for the different dose of TZP.

Compared with TZP 5 mg, meta-analysis revealed that TZP 10 mg increased total AEs by 6% (RR 1.06, 95% CI 1.01~1.11, *P* = 0.03) and GI AEs by 17% (RR 1.17, 95% CI 1.03~1.33, *P* = 0.02), while serious AEs (RR 0.94, 95% CI 0.74~1.19, *P* = 0.59), MACE-4 (RR 0.96, 95% CI 0.54~1.70, *P* = 0.88), cardiovascular death (RR 0.27, 95% CI 0.05~1.66, *P* = 0.16), hypertension (RR 0.71, 95% CI 0.32~1.58, *P* = 0.40), cancer (RR 0.42, 95% CI 0.06~2.83, *P* = 0.37), hypoglycaemia (PG < 70 mg/dl) (RR 1.22, 95% CI 1.00~1.48, *P* = 0.05) and hypoglycaemia (PG < 54 mg/dl) (RR 0.86, 95% CI 0.60~1.25, *P* = 0.44) were comparable. TSA showed these outcomes did not reach the RIS or TSA threshold, and the results need more research and demonstration. In comparison to TZP 5 mg, meta-analysis indicated that TZP 15 mg increased total AEs by 10% (RR 1.10, 95% CI 1.05 ~ 1.15, *P* = 0.0001), while serious AEs (RR 0.84, 95% CI 0.65~1.07, *P* = 0.16), GI AEs (RR 1.28, 95% CI 0.95~1.74, *P* = 0.11), MACE-4 (RR 0.59, 95% CI 0.30~1.14, *P* = 0.11), cardiovascular death (RR 0.79, 95% CI 0.21~2.91, *P* = 0.72), hypertension (RR 0.93, 95% CI 0.45~1.92, *P* = 0.84), cancer (RR 0.25, 95% CI 0.03~2.17, *P* = 0.21), hypoglycaemia (PG < 70 mg/dl) (RR 1.19, 95% CI 0.98~1.45, *P* = 0.08) and hypoglycaemia (PG < 54 mg/dl) (RR 1.07, 95% CI 0.75~1.51, *P* = 0.72) were comparable. TSA showed that cumulative Z values of total AEs exceeded the required information size at the fourth study (RIS = 2,216), suggesting that the risk observed with the current information size was conclusive. Meta-analysis demonstrated that total AEs (RR 1.04, 95% CI 0.99~1.09, *P* = 0.11), serious AEs (RR 0.89, 95% CI 0.70~1.15, *P* = 0.37), GI AEs (RR 1.01, 95% CI 0.90~1.14, *P* = 0.89), MACE-4 (RR 0.60, 95% CI 0.31~1.19, *P* = 0.15), cardiovascular death (RR 2.96, 95% CI 0.47~18.77, *P* = 0.25), hypertension (RR 1.28, 95% CI 0.58~2.85, *P* = 0.54), cancer (RR 0.33, 95% CI 0.01–8.03, *P* = 0.50), hypoglycaemia (PG < 70 mg/dl) (RR 0.98, 95% CI 0.81~1.18, *P* = 0.84) and hypoglycaemia (PG < 54 mg/dl) (RR 1.34, 95% CI 0.69~2.61, *P* = 0.38) were comparable for TZP 15 mg compared with TZP 10 mg. TSA showed that the results observed by the current information volume are not conclusive. The results of the meta-analysis, TSA, and quality of evidence are shown in Table 2. A comparison of each outcome for the different dose comparisons of TZP is shown in Figure 6.

**Table 2:** Meta-analysis and TSA results of different doses of TZP for AEs.

**Outcome**	**TZP arm (events/total)**	**Comparator arm (events/total)**	***I* ^2^**	**RR (95%CI)**	**TSA**	**RIS**	**Quality of evidence**
**TZP 10 mg vs. TZP 5 mg**							
Total AEs	1,013/1,448	958/1,449	0	1.06 (1.01, 1.11)	No	4,607	Moderate
Serious AEs	117/1,448	125/1,449	19	0.94 (0.74, 1.19)	No	116,856	Moderate
GI AEs	292/641	252/646	0	1.17 (1.03, 1.33)	No	1,792	Moderate
MACE-4	21/807	22/803	0	0.96 (0.54, 1.70)	No	416,265	Moderate
Cardiovascular death	1/797	5/799	17	0.27 (0.05, 1.66)	No	5,751	Moderate
Hypertension	10/530	14/529	0	0.71 (0.32, 1.58)	No	12,059	Moderate
Cancer	1/240	3/237	0	0.42 (0.06, 2.83)	No	3,641	Moderate
Hypoglycaemia (PG < 70 mg/dl)	137/651	111/650	25	1.22 (1.00, 1.48)	No	7,373	Moderate
Hypoglycaemia (PG < 54 mg/dl)	48/1,397	55/1,394	0	0.86 (0.60, 1.25)	No	42,953	Moderate
**TZP 15 mg vs. TZP 5 mg**							
Total AEs	1,062/1,461	958/1,449	25	1.10 (1.05, 1.15)	Yes	2,216	High
Serious AEs	106/1,461	125/1,449	0	0.84 (0.65, 1.07)	No	12,234	Moderate
GI AEs	296/644	252/646	71	1.28 (0.95, 1.74)	No	8,862	Low
MACE-4	13/817	22/803	0	0.59 (0.30, 1.14)	No	5,029	Moderate
Cardiovascular death	4/808	5/799	0	0.79 (0.21, 2.91)	No	104,369	Moderate
Hypertension	13/532	14/529	0	0.93 (0.45, 1.92)	No	21,984	Moderate
Cancer	0/241	3/237	0	0.25 (0.03, 2.17)	No	1,258	Moderate
Hypoglycaemia (PG <70 mg/dl)	135/653	111/650	47	1.19 (0.98, 1.45)	No	15,120	Moderate
Hypoglycaemia (PG <54 mg/dl)	60/1,408	55/1,394	0	1.07 (0.75, 1.51)	No	129,242	Moderate
**TZP 15 mg vs. TZP 10 mg**							
Total AEs	1,062/1,461	1,013/1,448	0	1.04 (0.99, 1.09)	No	8,616	Moderate
Serious AEs	106/1,461	117/1,448	0	0.89 (0.70, 1.15)	No	33,066	Moderate
GI AEs	296/644	292/641	18	1.01 (0.90, 1.14)	No	686,030	Moderate
MACE-4	13/817	21/807	0	0.60 (0.31, 1.19)	No	6,313	Moderate
Cardiovascular death	4/808	1/797	0	2.96 (0.47, 18.77)	No	7,202	Moderate
Hypertension	13/532	10/530	0	1.28 (0.58, 2.85)	No	21,984	Moderate
Cancer	0/241	1/240	0	0.33 (0.01, 8.03)	No	4,007	Moderate
Hypoglycaemia (PG < 70 mg/dl)	135/653	137/651	0	0.98 (0.81, 1.18)	No	378,528	Moderate
Hypoglycaemia (PG < 54 mg/dl)	60/1,408	48/1,397	57	1.34 (0.69, 2.61)	No	54,898	Low

### Publication bias assessment

**Figure 7:**
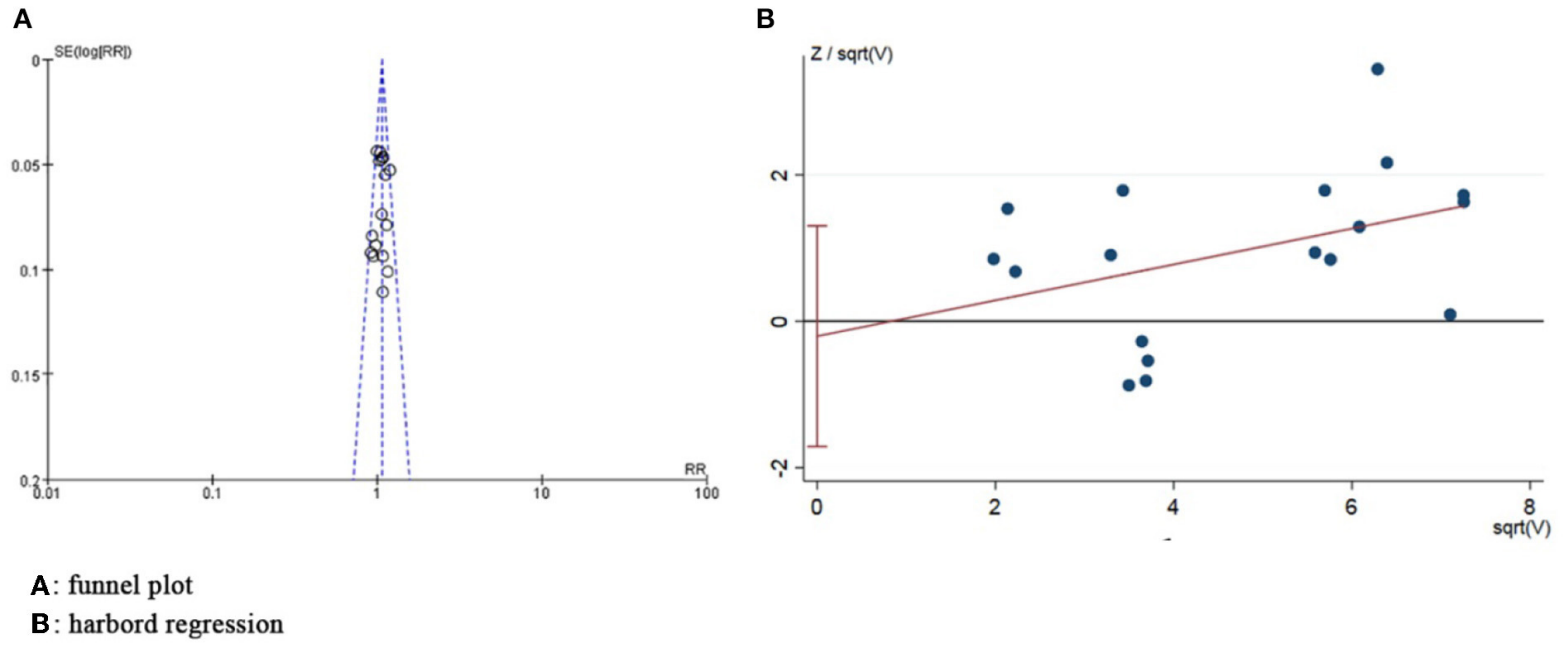
Publication bias assessment graph.

The funnel plot revealed the basic symmetry of the scatter on both sides, and Harbord regression of total AEs showed no significant publication bias (*P* = 0.78) (Figure 7).

## Discussion

Currently, metformin is still the first-line drug used for the initial treatment of T2DM (41), and it is the only hypoglycemic agent in the category of metformin that has received approval from the Food and Drug Administration (42). However, because of the poor glycemic control of metformin, it is often combined with other hypoglycemic agents (43). As T2DM progresses, the need of patients for glycemic control gradually increases, and most patients eventually require insulin for glycemic control (44). Glargine is the first long-acting basal analog of insulin with superior glucose-lowering effects (45, 46). But unfortunately, it increases the risk of weight gain and hypoglycemia (47), which are risk factors of T2DM, so the guidelines do not recommend insulin for all patients with T2DM (48). In recent years, GLP-1 RA has gradually been favored by clinicians and patients, and GLP-1 RA represented by semaglutide can effectively reduce glycemia and weight in patients with T2DM (49), with good clinical efficacy. However, there are concerns about GLP-1RA-induced GI AEs such as nausea, vomiting, and diarrhea (49, 50), and its efficacy remains limited in some patients with T2DM (49). As studies progress, novel hypoglycemic agents such as the novel mitochondrial modulator TRC150094 (51), the glucokinase activator dorzagliatin (52), and the GLP-1R and GIPR dual agonist TZP continue to be launched and show excellent hypoglycemic potential.

TZP is a novel GLP-1R and GIPR agonist. Previous meta-analyses have shown that TZP is significantly more effective than GLP-1RA and insulin in lowering glycemia and reducing weight, and does not increase total AEs or cardiovascular risk (14, 53), promising a new strategy for the treatment of T2DM. Meta-analyses published by Karagiannis et al. (20) and Bhagavathula et al. (21) comparing the efficacy and safety of TZP with placebo, insulin and GLP-1 RA for the treatment of T2DM confirmed that TZP was significantly more effective in lowering glycemia than placebo, insulin and GLP-1RA. Six clinical studies and a sample size of 4,358 were included in this meta-analysis and TSA, which is the first publication so far to study different doses of TZP in the treatment of T2DM. There are some differences in our study compared to Karagiannis et al. (20) and Bhagavathula et al. (21) (as shown below). (1) The research themes are different. While the theme of Karagiannis et al. (20) and Bhagavathula et al. (21) was the comparative efficacy and safety of TZP and other drugs such as placebo, insulin and GLP-1 RA, the theme of this study was the comparative efficacy and safety between different doses of TZP, with the aim of exploring the optimal dose and dose adjustment strategy of TZP. (2) The inclusion of studies and outcome indicators are enriched. Karagiannis et al. (20) explored efficacy endpoints such as HbA1c, FSG, and weight, as well as safety endpoints such as total AEs, GI AEs, serious AEs, and hypoglycaemia. However, they did not perform a detailed analysis of cardiovascular-related safety endpoints, and they included only 4 clinical studies due to search time limitations. Two additional relevant clinical studies became available after their study was published, and we included these new studies in our analysis. Bhagavathula et al. (21) included clinical studies published to date and compared the efficacy and safety of TZP with other drugs in terms of outcome indicators such as HbA1c, weight, hypoglycaemia, GI AEs, and serious AEs. Unfortunately, they did not conduct the analysis of FSG. Although HbA1c can reflect the average glycemic in the past 8–12 weeks, it is equally important to analyze FSG as an immediate glycemic indicator. Therefore, we carried out an analysis of FSG. We also performed analysis of safety endpoints such as total AEs, MACE-4, cardiovascular death, and hypertension to further understand the overall safety as well as cardiovascular risk of TZP. (3) The research methodology is innovated. On the one hand, we performed secondary evaluation for the results of meta-analysis by TSA, which allowed the credibility of the study results to be improved. On the other hand, we assessed publication bias by Harbord regression and introduced GRADE for evidence quality evaluation, which give a more comprehensive evaluation of this study. Overall, we used meta-analysis and TSA to explore the optimal dose and dose adjustment strategy for TZP, using dose as the core factor of this study.

### Efficacy analysis

Meta-analysis indicated that HbA1c, FSG and weight were significantly lower in the high-dose group of TZP than those in the low-dose group. TSA demonstrated that there was conclusive evidence for all efficacy endpoints except for the FSG of “TZP15 mg vs. TZP10 mg.” This implies that within the 5–15 mg dose, the effect of TZP in lowering glycemia and reducing weight is dose-dependent, and TZP 15 mg can achieve the best efficacy benefit in patients with T2DM. It is important to note that because of the presence of basal medication in 5 of the included studies (25–29), the benefit results we obtained are based on the context of the combination and not on the independent effect of TZP. It has been shown that the hypoglycemic effect of TZP is associated with increased insulin sensitivity in patients (30) and that the hypoglycemic effect of TZP gradually enhances with the increase of TZP dose. Both GLP-1 and gastric inhibitory polypeptide (GIP) have a facilitative effect on insulin secretion (16). TZP may reduce the metabolic demand for insulin secretion from pancreatic β-cells by decreasing insulin resistance in patients with T2DM, and subsequently reducing sustained ß-cell stress (31). And for some patients, the reduction in metabolic demand has the potential to reverse the dysfunction of islet β-cells (31). In addition, brown fat (BAT) plays a decisive role in the regulation of systemic glucolipid metabolism and can be a potential target for the treatment of T2DM (32). TZP can mediate its insulin-sensitizing effects in a weight-dependent and weight-independent manner by inducing metabolic pathways associated with glucose, free fatty acid (FFA) and branched-chain amino acid (BCAA) oxidation in BAT (33).

Being overweight or obese is a major risk factor for T2DM (34) and insulin resistance is more pronounced in obese patients with T2DM (35). Currently, guideline states that weight loss is one of the most effective therapies for the treatment of T2DM (36). TZP achieves dose-dependent weight loss through dual activation of GLP-1R and GIPR. GLP-1 has the effect of slowing gastric emptying and promoting satiety (12), and it also activates the anorexia pathway in the brain to suppress appetite in patients with T2DM (17). GIP, on the other hand, directly activates the GIPR located in the hypothalamus, which results in the suppression of food intake (37). The results of this study showed that TZP lowered glycemia and reduced weight in a dose-dependent manner. The reason may be that TZP at 5–15 mg failed to activate all GLP-1R and GIPR. Therefore, as the dose of TZP increases, the unsaturated GLP-1R and GIPR can continue to be activated, thus exerting a stronger effect on lowering glycemia and reducing weight. Rosenstock et al. (24) discovered that the effects of TZP on weight were persistent, implying that high-dose TZP can achieve sustained and effective weight loss. In addition, the hypolipidemic effect of TZP was also dose-dependent. Ruotolo et al. (38) revealed that high-dose TZP lowered total triacylglycerides (TAG), diacylglycerides (DAG), phosphatidylethanolamines (PE), phosphatidylcholines (PC), and phosphatidylinositols (PI) more significantly. Wilson et al. (19) observed that high-dose TZP had a better effect on lowering apoC-III, apoB, and triglyceride (TG).

### Safety analysis

In the safety analysis, the tables of AEs were relatively similar across the included studies. They accurately measured AEs such as hypoglycemia, hyperglycemia, hypertension, pancreatitis, and gallstones, but the incidence of these AEs was low and did not differ statistically. In terms of safety endpoints, meta-analysis showed no statistical difference in serious AEs, MACE-4, cardiovascular death, hypertension, cancer and hypoglycaemia between the three dose groups of TZP. This implies that serious AEs, cardiovascular AEs, and hypoglycemic events may not be related to the dose of TZP. Meta-analysis demonstrated no significant differences in total AEs, serious AEs, GI AEs, or hypoglycaemia between TZP 15 mg and TZP 10 mg, suggesting that the safety profiles of high-dose TZP and medium-dose TZP are comparable. Compared with TZP 5 mg, the total AEs is significantly increased in TZP 10 mg and TZP 15 mg, which means that low-dose TZP has better overall safety. It is important to emphasize that most of the included studies had basal medication and therefore the safety results are primarily based on the context of the combination and not on the independent effect of TZP. Interestingly, the results of this study showed a significant increase in GI AEs for TZP 10 mg compared to TZP 5 mg, while TZP 15 mg had comparable GI AEs. Sensitivity analysis displayed that the heterogeneity of “TZP 15 mg vs. TZP 5 mg” was derived from the study of Frias2018 (29). The heterogeneity disappeared after removing this study, and the effect sizes did not change significantly, while the results still suggested that the GI AEs were comparable between the two. Notably, the results of the studies (24, 28) showed that GI AEs were dose-independent, with the exception of Frias2018 (29) which supported a dose-dependent GI AEs for TZP, a discrepancy that may be related to the course of treatment. Frias2018 (29) was a study with a 26-week duration, and its results revealed a positive correlation between GI AEs of TZP and dose over a 6-month period. However, Rosenstock2021 (24) and Frias2021 (28) both found no significant correlation between GI AE and dose for a 40-week duration of TZP. This result is in agreement with Del Prato (26) who suggested that TZP-induced GI AEs decrease gradually over time (26), and therefore GI AEs of TZP in the long duration may not be significantly correlated with dose. In fact, GI AEs remain the most common AEs of TZP, but their severity is mostly mild (39) and decreases over time (26). The mechanism by which TZP causes GI AEs has not been fully elucidated and may be related to the activation of GLP-1R in the central nervous system (40), but no conclusive evidence is available.

### Limitations

Although this study strictly followed the PRISMA guidelines for systematic reviews and meta-analysis, it continues to have some limitations. The first aspect is that neither the Del2021 (26) nor the Ludvik2021 (27) studies were blinded to the intervention, increasing the risk of implementation bias in the included studies. But the implementation bias formed by the absence of blinding of personnel and participants in the Del2021 (26) and Ludvik2021 (27) studies was mainly present in the comparison between TZP and insulin, not between different doses of TZP. In fact, the two studies did not lead to methodological heterogeneity in the combined analysis, so we do not believe that the absence of blinding creates a serious bias in this study. In the second aspect, these studies have been conducted in a number of countries in North America, South America, Europe, Asia, and Oceania, but have not yet included countries with a predominantly African population. Therefore, the results of this study may be more favorable to European-Americans and Asians. This may be due to the limitations of the medical environment in Africa, and eventually the investigators did not set up research centers in Africa, and they may try to conduct TZP-related clinical studies in some safe and stable African countries with good medical environments in the future. The third aspect is that, five of the included studies (25–29) combined other hypoglycemic agents, so the results of the dose studies of TZP cannot be used to interpret the independent clinical efficacy and safety of TZP. In the fourth aspect, the lowest mean weight across the included studies was 85.4 kg. This suggests that the dose studies of TZP included mainly overweight or obese populations, and therefore it cannot clarify the effect of different doses of TZP in non-obese and non-overweight populations. The fifth aspect is the lack of sufficient short-term follow-up data. Although long-term efficacy is the key to measure the effect of different doses of treatment with TZP, short-term efficacy is of great significance due to the psychological urgency of patients with T2DM to lower their glycemia. All of the included studies were followed up for 26 weeks or more, and most of the efficacy dates obtained were for long-term outcomes, with a lack of short-term efficacy outcomes for TZP.

### Expectations

Given the limitations of the current study, future studies will be further improved. Firstly, increase the total sample size and use a rigorous randomized controlled double-blind trial. This not only improves the precision of the study, but also reduces potential methodological heterogeneity and increases the credibility of the results. Secondly, open research centers in countries with a predominantly people of African descent. This could explore the effects of TZP in populations of African descent and combine previous studies to synthesize the benefits and risks when using TZP in different ethnic groups. Thirdly, broaden the scope of efficacy indicators. 2-h postprandial glucose, lipids, blood pressure, body mass index (BMI), and cognitive function could be included as outcome indicators, which would allow a more comprehensive exploration of the combined benefits of TZP. Fourthly, control for relevant variables and perform a stratified study. A more comprehensive stratified analysis could be performed based on baseline information such as age, disease course, BMI, HbA1c level, and medication background, and thus learn about the efficacy and safety of TZP in different contexts. Fifthly, research in depth the properties of GIPR and the hypoglycemic potential of its activators. It has been shown that GIP stimulates insulin release to exert hypoglycemic effects under hyperglycemic conditions (28), while stimulating glucagon secretion to reduce the occurrence of hypoglycemia in hypoglycemic states (18). GIP also directly activates the hypothalamic GIPR thus effectively suppressing food intake (37), exerting a weight-reducing effect and enabling weight-independent sensitization to insulin (33). This seems to imply that GIPR may have a unique role in the treatment of T2DM and that its mechanistic effects and effects of action are valuable for further investigation.

There are also a number of TZP-related clinical studies currently in the planning or conducting stage. NCT04657003 is a multi-center clinical study. The study will be conducted in various countries and regions, including the United States, Brazil, Japan, India, Russia, Puerto Rico, and Taiwan, and it is planned to include a sample size of approximately 900 patients to explore the efficacy and safety of TZP for the treatment of patients with obese or overweight with T2DM when measured by weight, BMI, waist circumference, HbA1c, FSG, lipids, blood pressure, and fasting insulin. NCT04660643 is a multi-center clinical study involving more than 70 countries and regions with a similar study topic to NCT04657003 and is expected to be completed in May 2023. Interestingly, NCT05024032 will evaluate the efficacy and safety of TZP in non-T2DM patients who are obese or overweight with weight-related comorbidities, and this randomized, controlled, double-blind trial will be conducted in China and is expected to include a sample size of 210. NCT04184622 will study the role of TZP in weight maintenance or weight loss following a program of intensive lifestyle changes, and they plan to collect a sample size of 2539 in 170 countries and regions. The two studies focused on the weight-reducing effects of TZP, rather than the effect of hypoglycemia, and may help broaden the indications of TZP. We are looking forward to the publication of the data from these studies and expect that the findings will be of benefit to patients with T2DM and the populations with obesity.

## Conclusion

TZP 15 mg > TZP 10 mg > TZP 5 mg in terms of lowering glycemia and reducing weight. TZP 5 mg > TZP 10 mg = TZP 15 mg in terms of safety. On this basis, we recommend TZP 5 mg as the first-choice dose for patients with T2DM to minimize the risk of AEs while reducing glycemia and weight. If patients cannot effectively control their glycemia after taking TZP 5 mg, it is recommended to take TZP 15 mg directly to achieve the best effect of glycemic reduction. Nevertheless, the independent efficacy and safety at different doses of TZP remains to be tested, as most of the included studies have a background of basal medication.

## Data availability statement

The original contributions presented in the study are included in the article/supplementary material, further inquiries can be directed to the corresponding author.

## Author contributions

YY conceived and designed the study. GH and XY participated in data processing and statistical analysis. YY, GH, and SY drafted the manuscript. MZ, WJ, XY, and SY contributed to data analysis and interpretation. YY, MZ, and WJ supervised the review of the study. All authors seriously revised and approved the final manuscript.

## Funding

This work was supported by National Natural Science Foundation of China (81973753) and Natural Science Foundation of Hunan Province (2018JJ3405).

## Conflict of interest

The authors declare that the research was conducted in the absence of any commercial or financial relationships that could be construed as a potential conflict of interest.

## Publisher's note

All claims expressed in this article are solely those of the authors and do not necessarily represent those of their affiliated organizations, or those of the publisher, the editors and the reviewers. Any product that may be evaluated in this article, or claim that may be made by its manufacturer, is not guaranteed or endorsed by the publisher.
